# Integrative Analysis of Oxidative Stress and Cellular Senescence Pathways in Chronic Obstructive Pulmonary Disease

**DOI:** 10.3390/genes17060685

**Published:** 2026-06-10

**Authors:** Yanina Timasheva, Gulnaz Korytina, Vitaly Markelov, Timur Nasibullin, Leysan Akhmadishina, Yulia Aznabaeva, Shamil Zulkarneev, Olga Kochetova, Naufal Zagidullin

**Affiliations:** 1 Institute of Biochemistry and Genetics, Ufa Federal Research Centre of Russian Academy of Sciences, 450054 Ufa, Russia; guly_kory@mail.ru (G.K.); marckelow.vitalick2017@yandex.ru (V.M.); nasibullintr@yandex.ru (T.N.); leys82@mail.ru (L.A.); ovkochetova@bashgmu.ru (O.K.); 2 Department of Medical Genetics and Fundamental Medicine, Faculty of General Medicine, Bashkir State Medical University, 450008 Ufa, Russia; 3 Department of Biology, Faculty of Stomatology, Bashkir State Medical University, 450008 Ufa, Russia; 4 Laboratory of Cell Cultures of the Institute of Fundamental Medicine, Bashkir State Medical University, 450008 Ufa, Russia; 5 The Molecular Technology School, Ufa State Petroleum Technological University, 450064 Ufa, Russia; 6 Department of Propaedeutics of Internal Diseases, Faculty of General Medicine, Bashkir State Medical University, 450008 Ufa, Russia; 3251251@gmail.com (Y.A.); zulkarneev.shamil@gmail.com (S.Z.); znaufal@mail.ru (N.Z.)

**Keywords:** chronic obstructive pulmonary disease, polygenic scores, redox signalling, accelerated cellular senescence, long non-coding RNAs, genetic predictors

## Abstract

**Background/Objectives**: Chronic obstructive pulmonary disease (COPD) is increasingly viewed as a disorder of impaired cellular adaptation to chronic stress, involving oxidative injury, mitochondrial dysfunction, and accelerated cellular senescence. We investigated whether genetic variation in these pathways contributes to disease susceptibility, lung function impairment, and polygenic risk prediction. **Methods**: Thirty-three single-nucleotide variants were analysed in 747 patients with COPD and 703 controls. Associations with disease susceptibility and lung function parameters were assessed using regression models with correction for multiple testing. Weighted and unweighted polygenic scores were constructed from associated variants and evaluated using receiver operating characteristic and net reclassification improvement analyses. **Results**: Significant associations were identified in genes involved in antioxidant defence (*NFE2L2, HMOX1, GSR*), PI3K/AKT/mTOR signalling (*PIK3R1, PTEN*), mitochondrial function (*TOMM40*), cellular stress responses (*FOXO3A*), and long non-coding RNA regulation (*MEG3, CDKN2B-AS1*). The strongest association was observed for *PIK3R1* rs831125 (OR = 2.31, *p* = 2.53 × 10^−10^). Variants in *NFE2L2, PIK3R1, MEG3, MALAT1*, and *SIRT3* were additionally associated with pulmonary function parameters. The weighted polygenic score demonstrated good discriminative ability (AUC 68.8%, 95% CI 65.9–71.7%) and substantially improved prediction when combined with age, sex, and smoking exposure (AUC 88.1%, 95% CI 86.3–89.8%; NRI = 0.62, *p* = 2.21 × 10^−28^). **Conclusions**: The identified loci converge on interconnected pathways involved in cellular stress adaptation, mitochondrial homeostasis, and senescence, supporting their contribution to chronic obstructive pulmonary disease susceptibility and functional decline.

## 1. Introduction

Chronic obstructive pulmonary disease (COPD) is a progressive chronic disorder of the respiratory system characterized by persistent airway inflammation, small airway obstruction, and destruction of the lung parenchyma, primarily resulting from long-term exposure to cigarette smoke or other environmental pollutants [1,2]. COPD represents a leading cause of morbidity and mortality worldwide and imposes a substantial and growing economic and social burden. According to current projections, global mortality attributable to COPD is expected to exceed 5.4 million deaths annually by 2060, highlighting the urgent need for improved strategies for diagnosis, treatment, and prevention [3].

In high-income countries, COPD prevalence is now similar between men and women, reflecting the major role of smoking and the historical convergence of smoking patterns between sexes. In contrast, in low- and middle-income countries, non-smoking exposures such as household air pollution from biomass fuels play a major role, with a disproportionate impact on women, who are more heavily exposed [4]. Notably, COPD develops in only a subset of exposed individuals, with approximately one-third of smokers progressing to clinically significant disease, indicating a substantial contribution of genetic and epigenetic susceptibility factors [5]. Current evidence suggests that COPD arises from a complex interplay between genetic predisposition, epigenetic regulatory networks, and environmental exposures closely linked to lifestyle [6].

Despite extensive investigation of both molecular mechanisms and clinical features, the pathogenesis of COPD and the determinants of its phenotypic heterogeneity remain incompletely understood. Large-scale genomic studies have identified more than 20 susceptibility loci associated with COPD risk, lung function traits, and disease-related phenotypes, underscoring the polygenic nature of the disease [6,7,8,9]. These findings emphasize the need for integrative approaches combining genomics with other omics technologies to better characterize disease mechanisms and identify novel biomarkers [10].

A central concept in contemporary COPD pathogenesis is accelerated cellular senescence. Cellular senescence, a fundamental mechanism regulating tissue homeostasis, is driven by multiple interconnected processes, including DNA damage, telomere shortening, mitochondrial dysfunction, impaired autophagy, and disruption of proteostasis. The progressive airflow limitation observed in COPD reflects, at least in part, an acceleration of normal age-related decline in lung function, supporting the concept of COPD as a disease of premature lung ageing [11]. Indeed, hallmark features of ageing, such as telomere attrition, increased cellular senescence, and DNA damage, are similarly observed in the lungs of elderly individuals and patients with COPD [12].

Oxidative stress plays a central role in linking environmental exposure to cellular senescence. Reactive oxygen species (ROS), generated both by exogenous sources such as cigarette smoke and endogenous processes including inflammatory cell activation, contribute to cellular damage and dysregulated signalling [13]. Although ROS also function as essential intracellular signalling molecules, their excessive accumulation disrupts redox homeostasis and promotes chronic inflammation and tissue injury [14]. In COPD, increased levels of oxidative stress biomarkers have been detected in exhaled breath condensate, blood serum, and systemic circulation, reflecting both local and systemic oxidative imbalance. Reduced activity of the NRF2-mediated antioxidant defence system further exacerbates oxidative stress by impairing transcriptional regulation of cytoprotective genes [15]. Oxidative stress is closely linked to accelerated cellular senescence, as ROS-induced molecular damage accumulates over time, contributing to progressive functional decline. In COPD, oxidative stress exceeds that observed during normal ageing, promoting chronic inflammation, mitochondrial dysfunction, and degenerative changes [13]. These processes are further amplified by dysregulation of key signalling pathways, including PI3K/AKT/mTOR, which integrates cellular responses related to proliferation, metabolism, survival, and senescence [16].

COPD is increasingly recognized as a systemic disease with multiple comorbidities, including cardiovascular disease, type 2 diabetes, and metabolic syndrome. These conditions share common underlying mechanisms involving oxidative stress, chronic inflammation, and accelerated cellular ageing [17,18,19]. The accumulation of senescent cells and their associated secretory phenotype contributes to systemic inflammation and disease progression, further supporting the classification of COPD as an ageing-related disorder [20]. Regulatory pathways involving sirtuins, particularly SIRT1, have emerged as critical modulators of cellular senescence and oxidative stress responses. Reduced sirtuin activity has been implicated in accelerated lung ageing in COPD, affecting downstream signalling pathways such as SIRT1/FoxO3a and SIRT1/p53 [21]. Transcription factors of the FOXO family play a key role in regulating antioxidant defence, stress responses, apoptosis, and metabolism, as well as mitochondrial function and proteostasis [22,23]. Impairment of these regulatory networks contributes to cellular dysfunction and disease progression. In addition, non-coding RNAs, particularly long non-coding RNAs (lncRNAs), have emerged as important regulators of gene expression and cellular signalling. LncRNAs influence epigenetic regulation, RNA processing, and cell cycle control, and have been implicated in multiple pathological processes, including cellular senescence [24,25]. More than 2000 lncRNAs have been functionally associated with human diseases, highlighting their potential role in COPD pathogenesis [26].

Previous studies by our group have demonstrated the involvement of genes related to antioxidant defence, sirtuin signalling, PI3K/AKT pathways, FOXO transcription factors, and lncRNAs in COPD susceptibility and progression [27,28,29,30]. These findings underscore the importance of integrative genetic approaches and highlight the need for further comprehensive analysis.

Building on growing evidence linking oxidative stress, cellular senescence, and genetic susceptibility to COPD pathogenesis, the present study aimed to perform a comprehensive integrative analysis of single nucleotide variants in key regulatory genes involved in these processes. Specifically, we investigated 34 single nucleotide variants (SNVs) in genes associated with oxidative stress response, antioxidant defence, and cellular senescence pathways, including sirtuin signalling, FOXO transcription factors, PI3K/AKT/mTOR cascades, and lncRNAs, based on findings from our previous studies. In addition, we evaluated the predictive power of polygenic risk scores derived from COPD-associated variants, both independently and in combination with established clinical risk factors such as age, smoking status, smoking index, and sex. This approach was designed to improve understanding of the genetic architecture of COPD and to identify potential biomarkers for risk stratification and disease prediction within the framework of accelerated lung ageing.

## 2. Materials and Methods

### 2.1. Study Group

The present study was conducted in accordance with the principles of Good Clinical Practice and the Declaration of Helsinki of the World Medical Association for medical research involving human subjects. The study protocol was approved by the Ethics Committee of the Institute of Biochemistry and Genetics of the Ufa Federal Research Centre of the Russian Academy of Sciences (Ufa, Russian Federation) (Protocol No. 19, 1 November 2022). Written informed consent was obtained from all participants prior to enrolment and collection of biological material. All DNA samples included in the study were anonymised.

Participant recruitment and sample collection were carried out between November 2022 and December 2025. Patients with COPD were consecutively recruited from the Department of Pulmonology of City Clinical Hospital No. 21 and the Department of Thoracic Surgery of the University Clinic of Bashkir State Medical University (Ufa, Russian Federation). Identical inclusion and exclusion criteria, clinical assessment procedures, sample collection protocols, and genotyping workflows were applied at both centres to ensure methodological consistency.

The study group comprised 1450 unrelated individuals, including 747 patients with COPD and 703 healthy controls. To minimise the risk of population stratification and false-positive genetic associations, all participants were ethnically homogeneous and self-identified as Tatars. Ethnic ancestry up to the third generation was verified through direct interviews with all study participants.

COPD diagnosis was established in accordance with the International Classification of Diseases, 10th Revision (ICD-10 http://www.who.int/classifications/icd/en/, accessed on 22 May 2026) and the recommendations of the Global Initiative for Chronic Obstructive Lung Disease [31]. Inclusion and exclusion criteria, as well as the methods of instrumental examination, have been described previously [27,28,29,30,32]. The diagnosis of COPD was based on a comprehensive assessment of clinical presentation, laboratory findings, and instrumental investigations, including high-resolution computed tomography and spirometry. Eligible patients demonstrated persistent irreversible airflow limitation, defined as post-bronchodilator forced expiratory volume in one second (FEV1) < 80% of the predicted value and a post-bronchodilator FEV1/forced vital capacity (FVC) ratio < 70%. COPD severity was classified according to GOLD criteria based on post-bronchodilator FEV1 values [31] (https://goldcopd.org). To provide a comprehensive assessment of disease severity and symptom burden, validated clinical instruments were applied. The COPD Assessment Test (CAT) was used to quantify symptom severity and the impact of COPD on daily activities, whereas the Modified Medical Research Council Dyspnoea Scale (mMRC) was used to assess the severity of dyspnoea [31].

Control subjects were recruited from individuals undergoing routine medical examinations at both participating centres. The control group consisted of unrelated individuals with no history of chronic disease, including respiratory disorders, and without symptoms of acute respiratory infection at the time of blood sampling. All control participants underwent clinical and instrumental examination, including pulmonary function testing with assessment of forced expiratory volume (FEV), FEV1, and the FEV1/FEV ratio. Inclusion criteria for the control group included normal pulmonary function (FEV1/FEV > 70% and FEV1 > 80%) and age over 45 years.

### 2.2. Genes and SNV Selection

The study employed a case-control design focused on genes involved in signalling pathways regulating oxidative stress, cellular senescence, apoptosis, mitochondrial dysfunction, regulatory non-coding RNAs, and the progression of age-related diseases. Candidate genes and biological pathways were identified using publicly available databases and literature resources, including Google Scholar (https://scholar.google.com/), GeneCards (https://www.genecards.org/), and KEGG (Kyoto Encyclopedia of Genes and Genomes; https://www.genome.jp/kegg/, accessed on 22 May 2026).

The most extensively studied single nucleotide variants (SNVs) within these genes were identified using the GWAS Catalog (https://www.ebi.ac.uk/gwas/, accessed on 22 May 2026), NCBI (https://www.ncbi.nlm.nih.gov/), and Ensembl (https://www.ensembl.org/index.html (accessed on 22 May 2026). Selection of functionally relevant single nucleotide variants (SNVs) was based on their potential effects on gene expression and regulatory activity in tissues and cell types implicated in COPD pathogenesis. Functional annotation and prioritisation of SNVs were performed using RegulomeDB v1.1 (https://regulomedb.org), SNPinfo Web Server—SNP Function Prediction (FuncPred) (https://snpinfo.niehs.nih.gov/), HaploReg v4.1 (https://pubs.broadinstitute.org/mammals/haploreg/haploreg.php, accessed on 22 May 2026), PolyPhen-2 (http://genetics.bwh.harvard.edu/pph2/, accessed on 22 May 2026), the Genotype-Tissue Expression (GTEx) project (https://www.gtexportal.org/, accessed on 22 May 2026), and eQTLGen (https://eqtlgen.org/, accessed on 22 May 2026). Potential interactions between SNVs and microRNAs were evaluated using miRBase (http://www.mirbase.org/, accessed on 22 May 2026). An additional selection criterion was a minor allele frequency (MAF) ≥ 5% in European populations. Detailed information on all selected genes and SNVs is provided in Supplementary Table S1.

### 2.3. Genotyping and Quality Control

Peripheral venous blood samples (4 mL) were collected from each participant into EDTA-containing tubes and stored at −4 °C prior to DNA isolation. Genomic DNA was extracted using the standard phenol–chloroform method [33]. Allelic discrimination was performed using real-time polymerase chain reaction (PCR) on a Bio-Rad CFX96 system (Bio-Rad Laboratories Inc., Hercules, CA, USA) with TaqMan SNP genotyping assays (Thermo Fisher Scientific Inc., Waltham, MA, USA, https://www.thermofisher.com/), PCR-restriction fragment length polymorphism (PCR-RFLP) analysis, or allele-specific PCR using the C1000 Touch thermal cycler (Bio-Rad Laboratories Inc., Hercules, CA, USA). Detailed information regarding genotyping assays and analytical methods for each SNV is presented in Supplementary Table S2.

Comprehensive descriptions of the genotyping procedures have been reported previously [27,28,29,30,32], as well as in earlier studies [34,35,36,37,38].

### 2.4. Power Analysis

Statistical power calculations were performed using the online Genetic Association Study Power Calculator (http://csg.sph.umich.edu/, accessed on 22 May 2026). The following parameters were used: disease prevalence of 0.141 according to epidemiological studies conducted in the Russian Federation [39,40], significance level *p* < 0.05, genotype relative risk of 1.5, minimum minor allele frequency (MAF) of 0.04 (rs2536), and an additive genetic model. Power estimates calculated for each investigated SNV are presented in Supplementary Table S3.

### 2.5. Statistical Association Analysis

Statistical analyses were performed using PLINK v2.0 (www.cog-genomics.org/plink/2.0/, accessed on 22 May 2026) [41]. Hardy–Weinberg equilibrium was assessed for all variants, and loci deviating from equilibrium were excluded from subsequent analyses. Associations between genetic variants and COPD susceptibility were evaluated using logistic regression under an additive genetic model with adjustment for sex. Under this model, the effect size is assumed to increase proportionally with the number of risk alleles carried.

Associations with quantitative phenotypes were analysed using generalised linear models adjusted for age, sex, and smoking exposure expressed as pack-years. To account for multiple comparisons, false discovery rate (FDR) correction was performed using the Benjamini–Hochberg procedure [42], and adjusted P_FDR_ values below 0.05 were considered statistically significant.

### 2.6. Polygenic Score Calculation

Both weighted and unweighted polygenic scores were calculated using genetic variants demonstrating significant associations with COPD in logistic regression analyses. Odds ratios estimated for each variant were used as weights for the corresponding risk alleles. For variants with odds ratios below 1.0, the alternative allele was designated as the effect allele. Variants located on the same chromosome were additionally assessed for linkage disequilibrium. In cases of significant linkage disequilibrium, only one representative variant was retained for inclusion in polygenic score calculations.

### 2.7. Receiver Operating Characteristic Analysis

Predictive models for COPD were constructed using the Epi: Statistical Analysis in Epidemiology and pROC packages implemented in the R statistical environment. Receiver operating characteristic (ROC) analysis was performed to evaluate the discriminative performance of the derived polygenic scores. Model performance was assessed using the area under the ROC curve (AUC), reflecting the ability of the classifier to distinguish between COPD cases and controls. Predictive performance was interpreted according to the following criteria: poor (AUC 0.50–0.60), satisfactory (0.60–0.70), good (0.70–0.80), very good (0.80–0.90), and excellent (>0.90).

### 2.8. Net Reclassification Improvement Analysis

The added predictive value of the polygenic scores was assessed using net reclassification improvement (NRI) [43]. The model including age, sex, and smoking index was used as the reference model and was compared with models additionally including either the unweighted or weighted polygenic score. NRI was calculated using the nribin function from the R package nricens, designed for risk prediction models with binary outcomes [44]. Bootstrapping was used to estimate 95% confidence intervals for NRI.

## 3. Results

### 3.1. Study Population Characteristics

The study included 1450 unrelated individuals, comprising 747 patients with COPD and 703 healthy controls. The demographic and clinical characteristics of the study population are presented in Table 1. COPD patients demonstrated the expected reductions in pulmonary function and higher symptom burden compared with controls, confirming the clinical distinction between the study groups.

### 3.2. Results of the Association Analysis

Associations between 33 genetic variants and COPD were evaluated using logistic regression under the additive model with adjustment for sex (Supplementary Table S4). Eleven SNVs remained significantly associated with COPD after correction for multiple testing (Table 2).

Next, we assessed the associations between these SNVs and quantitative phenotypes (Supplementary Table S5). The results showed that *NFE2L2* rs6721961*G allele was associated with an increased risk of COPD (OR = 1.39, P_FDR_ = 0.002), and with reduced pulmonary function parameters, including VC (β = −5.51, P_FDR_ = 0.004), and FVC (β = −6.73, P_FDR_ = 0.001) (Table 3). *PIK3R1* rs831125*A allele was associated with an increased risk of COPD (OR = 2.22, P_FDR_ = 4.05 × 10^−10^) and with decreased FVC (β = −7.34, P_FDR_ = 0.042). In addition, *MEG3* rs7158663*G allele was associated with COPD (OR = 1.72, P_FDR_ = 1.67 × 10^−8^), whereas *MEG3* rs7158663*A allele was associated with higher FVC (β = 6.50, P_FDR_ = 0.025). *SIRT3* rs3782116*G allele was associated with VC (β = 4.11, P_FDR_ = 0.049) and *MALAT1* rs619586*A was associated with FEV1/FVC (β = 8.74, P_FDR_ = 0.035), although neither showed a statistically significant association with COPD.

### 3.3. Polygenic Score Analysis

**Figure 1:**
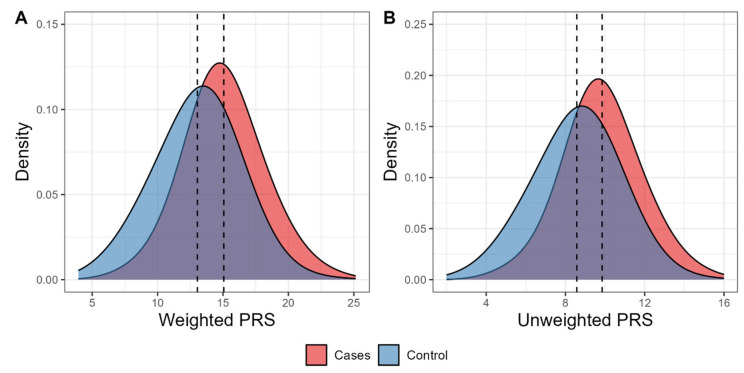
Density plots showing the distribution of polygenic scores. (**A**) Distribution of weighted scores in controls (blue) and individuals with COPD (red). (**B**) Distribution of unweighted scores in controls (blue) and individuals with COPD (red), The dotted lines represent mean values for each group.

Polygenic score analysis assumes an additive contribution of the alleles [45]. Polygenic scores were calculated using odds ratios (ORs) obtained for variants significantly associated with COPD in the regression analysis as weights. Figure 1 presents the distributions of weighted and unweighted polygenic scores in COPD cases and controls. Mean values of both scores were higher in individuals with COPD compared to controls (15.06 ± 0.10 vs. 13.04 ± 0.11 or weighted scores; 9.85 ± 0.07 vs. 8.57 ± 0.07 for unweighted scores). Analysis of weighted polygenic scores showed that the combined effect of the 11 SNVs was associated with increased disease risk (OR [95% CI OR] = 1.28 [1.23–1.34], P = 2.84 × 10^−32^). Similar results were obtained for unweighted scores, indicating an association between the joint effect of these variants and elevated COPD risk (OR [95% CI OR] = 1.43 [1.35–1.52], P = 3.31 × 10^−30^).

**Figure 2:**
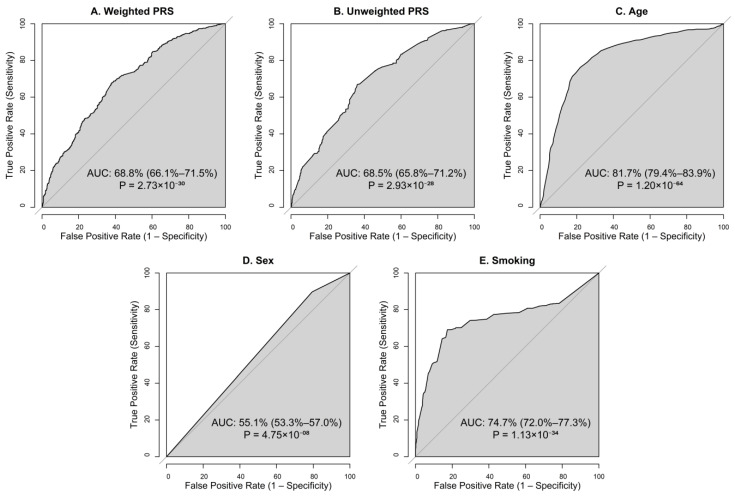
Receiver operating characteristic (ROC) curves illustrating model performance for COPD prediction. (**A**) Weighted polygenic score (11 variants). (**B**) Unweighted polygenic score (11 variants). (**C**) Age. (**D**) Sex. (**E**) Smoking (pack-years). Sensitivity represents the true positive rate, and specificity the true negative rate. AUC reflects model performance: ≥90% excellent, 80–90% very good, 70–80% good, 60–70% satisfactory, and 50–60% poor.

We used the Receiver Operating Characteristic (ROC) analysis to evaluate the predictive performance of the constructed models based on weighted and unweighted polygenic scores, as well as clinical variables (age, sex, and smoking [pack-years]). The performance of single-predictor models is shown in Figure 2. The highest area under the curve (AUC) was observed for age (AUC 81.7%, 95% CI 79.4–83.9%), and the lowest for sex (AUC 55.1%, 95% CI 53.3–57.0%). The model based on the weighted polygenic score (11 SNVs) yielded an AUC of 68.8% (95% CI 66.1–71.5%) (Figure 2), indicating moderate discriminative ability.

**Figure 3:**
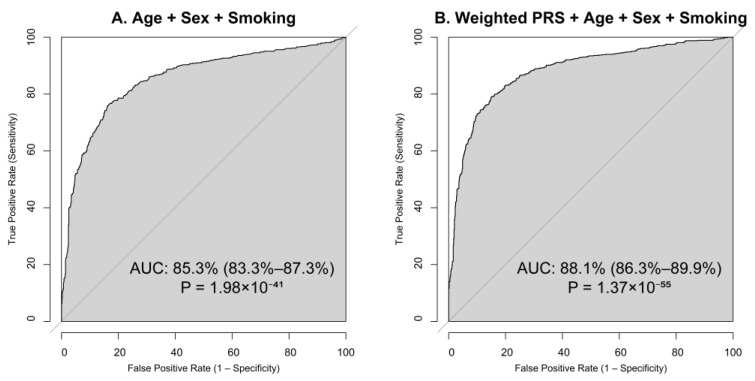
Receiver operating characteristic (ROC) curves for combined models predicting COPD. (**A**) Model including age, sex, and smoking (pack-years). (**B**) Model including weighted polygenic score (11 variants) combined with age, sex, and smoking (pack-years).

We further evaluated models combining non-genetic risk factors with and without the weighted polygenic score (Figure 3). The model incorporating polygenic score, age, sex, and smoking showed the highest predictive performance (AUC 88.1%, 95% CI 86.3–89.9%).

NRI analysis was performed using the non-genetic model, including age, sex and smoking index, as the reference model. The reference model was compared with models additionally including either the unweighted or weighted polygenic score (Table 4). Both scores significantly improved reclassification. The unweighted score showed a slightly higher total NRI than the weighted score (NRI = 0.64, P = 2.92 × 10^−21^ vs. NRI = 0.62, P = 2.21 × 10^−28^, respectively), mainly because of better reclassification of controls (NRI = 0.34, P = 3.15 × 10^−25^ vs. NRI = 0.30, P = 7.47 × 10^−20^, respectively). By contrast, the weighted score showed slightly better reclassification of COPD patients (NRI = 0.32, P = 3.50 × 10^−19^ vs. NRI = 0.30, 4.40 × 10^−8^, respectively).

## 4. Discussion

COPD is a complex multifactorial disease resulting from interactions between environmental exposures, ageing-related procedures, and genetic susceptibility. In the present study, we identified significant associations between COPD and genetic variants involved in oxidative stress responses, cellular senescence, PI3K/AKT/mTOR signalling, FOXO-mediated stress responses, mitochondrial function, and lncRNA-dependent regulation. We also demonstrated that polygenic scores derived from these variants improved COPD risk prediction when combined with established risk factors, including age, sex, and smoking exposure. In addition, several variants were associated with quantitative measures of lung function, supporting their potential contribution to disease severity and functional decline.

These findings are consistent with the current understanding of COPD as a disease of accelerated lung ageing characterised by impaired stress responses, mitochondrial dysfunction, chronic inflammation, and defective tissue repair [1,11,14,15,17,20]. Although long-term cigarette smoking remains the major environmental risk factor, only a subset of smokers develop clinically significant airflow limitation, indicating that inherited susceptibility modifies individual responses to chronic inhalational injury [3,4,5,6]. The development of COPD is therefore best understood as the result of long-term interaction between genetic predisposition, environmental exposures, and ageing-related biological processes. Identification of molecular mechanisms contributing to early disease development is particularly important, as these mechanisms may help explain why some individuals are more vulnerable to progressive airflow limitation than others [46,47].

Our results suggest that genetic variation affecting the maintenance of redox homeostasis, mitochondrial integrity, and cellular resilience may contribute to this inter-individual variability. COPD pathogenesis involves multiple interacting molecular pathways that promote airway obstruction, parenchymal damage, and remodelling of the respiratory tract [46]. Chronic exposure to cigarette smoke and other inhaled toxicants increases the production of reactive oxygen species (ROS), leading to oxidative damage to cell membranes, proteins, nuclear DNA, and mitochondrial DNA [48,49]. Mitochondrial dysfunction further amplifies ROS generation through disruption of oxidative phosphorylation and mitophagy [50]. Damage to mitochondria, membranes and proteins promotes stress-induced premature senescence of airway epithelial and alveolar cells through activation of p53/p21 and p16 pathways [51]. This leads to irreversible cell-cycle arrest and accumulation of cells with a senescence-associated secretory phenotype (SASP), which further sustains local and systemic inflammation [52]. The association signals identified in the present study within genes involved in oxidative stress regulation, mitochondrial homeostasis, chronic inflammation, and cellular senescence provide additional support for the importance of these mechanisms in COPD susceptibility and progression.

The associations observed for *NFE2L2***,** *HMOX1,* and *GSR* highlight the importance of antioxidant defence pathways in COPD. Chronic exposure to cigarette smoke and activation of inflammatory cells increases the oxidant burden in the airways and lung parenchyma. Under these conditions, genetic variation affecting antioxidant transcriptional programmes, haem degradation or glutathione recycling may influence the capacity of lung tissue to counteract oxidative injury.

The NRF2/KEAP1 pathway occupies a central position among redox-sensitive cellular systems. It activates gene expression through the interaction of the NRF2 transcription factor with antioxidant response elements (AREs) in the promoter regions of target genes [53]. *NFE2L2*, located at 2q31.2, encodes NRF2, a tightly regulated transcription factor that coordinates the cellular stress response [54,55]. NRF2 binds antioxidant response elements in the promoter regions of target genes and activates a broad transcriptional programme involved in detoxification, antioxidant defence and maintenance of redox homeostasis [56,57]. Its target genes include *HMOX1, GSR**,*** glutathione S-transferases, and *NQO1*, among many others [58]. NRF2 also modulates inflammation, autophagy, apoptosis, pyroptosis and ferroptosis, reflecting its pleiotropic role in cellular adaptation to stress [59,60]. Given the continuous exposure of lung tissue to oxidants and xenobiotics, variation affecting NRF2 expression, translocation or binding to antioxidant response elements may be particularly relevant to COPD susceptibility [61]. Several *NFE2L2* polymorphisms have been associated with reduced gene expression, impaired antioxidant responses and lower FEV1, supporting the relevance of this pathway to airflow limitation [62,63,64]. Reduced *NFE2L2* expression has also been reported in lung macrophages from elderly smokers and patients with COPD, as well as in peripheral blood mononuclear cells from COPD patients [65,66]. A number of studies have also implicated *NFE2L2* SNVs in atherosclerosis, bronchial asthma, COPD and ageing-related processes, further supporting the broader relevance of NRF2-mediated stress-response pathways to chronic inflammatory and age-associated diseases [67,68,69,70,71,72,73].

*HMOX1* encodes haem oxygenase-1, an inducible enzyme that degrades free haem into biliverdin, carbon monoxide and ferrous iron. Through this activity, HMOX1 contributes to cytoprotection, limits oxidative stress and inflammation, and modulates mitochondrial injury and DNA damage [74,75,76]. In chronic inflammatory conditions, *HMOX1* expression increases as part of the adaptive stress response and is regulated by redox-sensitive transcription factors, including NRF2, AP-1, HIF, NF-κB and Bach1 [77,78]. The role of HMOX1 in COPD is complex and may depend on cell type and inflammatory context. In epithelial cells and lung fibroblasts, activation of the NRF2/HMOX1 axis may suppress senescence and reduce SASP-associated inflammatory signalling [79,80]. In contrast, excessive HMOX1 activity in alveolar macrophages may increase intracellular iron release, promote Fenton chemistry and contribute to lipid peroxidation and ferroptosis if iron sequestration is insufficient [81,82]. The best-studied COPD-associated variation in *HMOX1* is the length polymorphism of GT repeats in the 5′ promoter region, where shorter repeats are generally associated with higher transcriptional activity and a stronger antioxidant response [83,84]. The variant analysed in the present study, rs2071749, is located in intron 3 and has been reported to be in linkage disequilibrium with promoter variants affecting transcription-factor binding, providing a plausible regulatory mechanism [85].

*GSR* encodes glutathione reductase, an NADPH-dependent flavoprotein that reduces oxidised glutathione to reduced glutathione, the major intracellular thiol antioxidant [86]. GSR activity is required to maintain the glutathione pool, support glutathione peroxidase function, neutralise ROS and reactive nitrogen species, and regulate redox signalling in lung epithelial and immune cells [87]. Disturbances in glutathione homeostasis have been described in COPD and are linked to oxidative injury, impaired antimicrobial defence and steroid resistance [88]. Previous work has associated *GSR* rs1002149 with COPD in non-smoking individuals [28], and experimental suppression of GSR has been shown to increase ROS accumulation and activate TGF-β/Smad signalling [87]. In addition, expression profiling of glutathione-metabolism genes in peripheral blood mononuclear cells from patients with COPD showed that higher GSR expression correlated with poorer lung function [89]. These observations support the biological plausibility of the association between *GSR* variation and COPD-related phenotypes.

The associations of *PIK3R1* and *PTEN* variants with COPD point to the involvement of PI3K/AKT/mTOR signalling, a pathway that integrates inflammatory, metabolic, survival and senescence-related signals [16]. Dysregulation of this pathway may contribute to impaired epithelial repair, altered autophagy, mitochondrial dysfunction and persistence of senescent cells [90]. *PIK3R1* encodes the regulatory subunit 1 of phosphoinositide 3-kinase and has been implicated in metabolic disease, cancer and ageing-related processes [37,91,92]. Recent evidence suggests that PIK3R1 may be involved in the regulation of COPD-associated ageing mechanisms [93]. The associations of *PIK3R1* variants with both COPD and lung function traits suggest that inherited differences in PI3K pathway regulation may modify cellular responses to smoke-induced stress and tissue remodelling [27,90].

PTEN is a key negative regulator of PI3K signalling and participates in the control of cell proliferation, apoptosis, inflammation, transcriptional regulation and genomic stability [94,95]. Reduced PTEN activity leads to activation of PI3K/AKT signalling and may promote cellular senescence [11]. Variation in *PTEN* has previously been investigated in COPD and other respiratory diseases, including asthma, pulmonary hypertension and idiopathic pulmonary fibrosis [95,96,97]. Mechanistically, PI3K/AKT/mTOR signalling may contribute to COPD through regulation of autophagy–lysosomal pathways, proteostasis, ribosome biogenesis, mitochondrial metabolism and maintenance of the SASP [98,99]. Increased PI3K activity has been reported in lung cells from patients with COPD, while PTEN and SHIP1, both of which contain oxidation-sensitive cysteine residues, provide redox-sensitive control of PI3K activity [94,100]. These observations link oxidative stress directly to dysregulation of PI3K/AKT/mTOR signalling in COPD [101].

The association of *FOXO3A* is consistent with the role of FOXO transcription factors in antioxidant defence, apoptosis, autophagy, mitochondrial function, metabolism and longevity-related pathways [22,23,29]. As a downstream target of AKT signalling, FOXO3 also connects PI3K/AKT pathway activity with cellular ageing mechanisms [22]. In COPD, disruption of these pathways may favour mitochondrial dysfunction, defective stress adaptation, and accumulation of senescent cells, consistent with the concept of COPD as a disease with features of accelerated lung ageing [23,29].

Variation in *FOXO3A* has been linked to longevity and age-associated diseases, placing this locus among genetic determinants of ageing-related stress responses [102,103,104,105,106,107]. FOXO transcription factors coordinate cellular adaptation to stress by activating genes involved in antioxidant defence and DNA repair. They also regulate the G1/S cell-cycle transition through induction of cyclin-dependent kinase inhibitors p27 (CDKN1B) and p21 (CDKN1A), as well as RBL2, reducing expression of E2F target genes and blocking cell-cycle progression [108]. Their role in cellular ageing also involves regulation of proteostasis, including induction of heat-shock proteins in response to mitochondrial stress [109,110].

FOXO activity is closely linked to sirtuin-mediated regulation. FOXO1 and FOXO3 are key substrates of NAD+-dependent sirtuin deacetylases, including SIRT1, SIRT2, SIRT3, and SIRT6. SIRT1-mediated deacetylation of FOXO1 stimulates its transcriptional activity and promotes autophagy [111], while SIRT1 also enhances FOXO3 binding to the *MAP1LC3B/LC3B* promoter and contributes to regulation of cellular senescence [112]. SIRT6 directly deacetylates FOXO1, increasing *FOXO1* transcriptional activity, promoting autophagy and counteracting cellular senescence [113,114]. SIRT3-mediated deacetylation of FOXO3A activates expression of catalase and mitochondrial superoxide dismutase 2 (SOD2), thereby reducing reactive oxygen species levels [115].

In lung epithelial cells, FOXO3 participates in mitochondrial quality control and the response to cigarette-smoke-induced injury. *FOXO3* transcriptional activity limits excessive cigarette-smoke-induced senescence in COPD lung epithelium [116,117] and regulates mitochondrial homeostasis and mitophagy, a key adaptive response to oxidative stress [118]. Increased acetylation of FOXO3 reduces PTEN-induced kinase 1 (PINK1) levels, suppresses mitophagy and promotes mitochondrial-damage-associated cellular senescence after cigarette-smoke exposure [119]. Accumulation of damaged mitochondria due to impaired SIRT1/FOXO3 signalling may then amplify oxidative stress and proteostatic dysfunction [120].

The effects of FOXO3 in COPD-related pathways appear to depend on cellular context and disease stage. Disruption of SIRT1-mediated FOXO3 deacetylation in cigarette-smoke-exposed lung cells prevents FOXO3 interaction with the survivin promoter, increases survivin expression, and inhibits apoptosis [121]. This may impair clearance of senescent cells and promote accumulation of cells with a senescence-associated secretory phenotype [122]. At the same time, FOXO3 activity supports epithelial-cell survival and suppresses inflammatory cytokines such as IL-6 and IL-8 [123]. Conversely, altered PI3K/AKT-mediated phosphorylation of FOXO3 in alveolar epithelial cells may promote apoptosis [124], and FOXO3 can induce expression of the pro-apoptotic mediator BIM, potentially contributing to alveolar epithelial-cell loss and emphysema development [125].

Taken together, these mechanisms place FOXO3A within a network linking PI3K/AKT signalling, sirtuin activity, mitochondrial quality control, oxidative stress, senescence and apoptosis in COPD. Deacetylated FOXO proteins support mitochondrial function, reduce oxidative stress and counteract senescence of lung epithelium [126]. The antioxidant function of FOXO3 is mediated in part through increased expression of SOD2, CAT and GPX1, which regulate reactive oxygen species homeostasis in lung cells under oxidative stress [127].

The association of *TOMM40* further supports the contribution of mitochondrial pathways to COPD pathogenesis. *TOMM40* encodes a subunit of the translocase of the outer mitochondrial membrane complex, which is required for mitochondrial protein import, mitochondrial biogenesis and maintenance of respiratory-chain function. Disruption of TOMM40 expression or function can impair protein import, disturb cellular respiration, increase mitochondrial reactive oxygen species production, and induce mitophagy or apoptosis [128]. In cellular models, TOMM40 dysfunction has been associated with pronounced mitochondrial impairment and increased oxidative damage, linking this gene to mechanisms of cellular ageing that are relevant both to COPD and to systemic age-associated diseases [129]. *TOMM40* variants may also modify susceptibility to mitochondrial dysfunction induced by oxidative stress [129]. In GWAS, *TOMM40* rs2075650 has been associated with longevity in older age, inflammatory responses and increased vulnerability to cardiovascular risk factors [128,130,131,132]. Its proximity to *APOE* places this locus within a genomic region repeatedly implicated in ageing-related disease, although further work is needed to determine whether the observed association reflects *TOMM40*, *APOE*, or another linked signal.

The associations involving *MEG3* and *CDKN2B-AS1* support the emerging role of lncRNA-mediated regulation in COPD. LncRNAs can influence epigenetic regulation, cell-cycle control, inflammation, apoptosis, and senescence, and previous work has implicated polymorphic loci in lncRNA genes in COPD susceptibility [24,25,26,30]. The 9p21 region containing *CDKN2B-AS1* is of particular interest because of its established role in ageing-related and cardiovascular phenotypes, which are clinically relevant in COPD [133,134]. *CDKN2B-AS1* rs4977574 may interfere with normal CDKN2B-AS1 function by altering splicing activity and downstream expression of genes involved in cell-cycle regulation, including p15/CDKN2B [135]. This variant has also been associated with atheromatosis in different vascular beds, while atheromatosis is common in COPD and has been linked to the emphysematous phenotype [136,137,138].

Experimental data further connect CDKN2B-AS1 with processes relevant to COPD pathobiology. Suppression of CDKN2B-AS1 increases the viability of human bronchial epithelial cells by reducing apoptosis and inflammation [139]. In vascular smooth-muscle cells, CDKN2B-AS1 may support cell viability through regulation of the miR-181a/SIRT1 axis [140]. It has also been implicated in inflammatory signalling, cellular senescence and apoptosis [25,141,142]. Mechanistically, CDKN2B-AS1 may interact with PI3K/AKT/mTOR signalling through binding to polycomb repressive complex proteins, regulation of the let-7c-5p/NAP1L1 axis and EZH2-mediated epigenetic silencing of *PTEN* [143,144]. These links place CDKN2B-AS1 at the intersection of lncRNA regulation, senescence, vascular pathology, and intracellular signalling pathways relevant to COPD.

MEG3 provides another example of lncRNA-mediated regulation potentially relevant to COPD. MEG3 participates in apoptosis through activation of p53 and inhibition of Bcl-xL, and its overexpression has been associated with reduced TGF-β1 levels, lower PI3K/AKT pathway activity and suppression of epithelial–mesenchymal transition [145,146]. It also interacts with TGF-β/SMAD3 and Wnt signalling pathways [147]. Beyond COPD-related mechanisms, MEG3 has been implicated in cancer, type 2 diabetes, acute lymphoblastic leukaemia, and osteoarthritis, supporting its broader role in proliferative, metabolic, and ageing-related disease processes [148,149,150,151].

In the lung, MEG3 may be particularly relevant through its links with senescence, oxidative stress, and apoptosis [152,153]. A MEG3-related senescence axis involving miR-125a-5p and the Sp1/SIRT1/HIF-1α pathway suggests that MEG3 may reduce senescence of lung epithelial cells by inhibiting miR-125a-5p [154]. MEG3 also regulates miR-664a-3p, while the miR-664a-3p/FHL1 axis has been linked to cigarette-smoke-induced oxidative stress, with miR-664a-3p expression negatively correlated with lung function [155]. In addition, MEG3 may regulate apoptosis through interactions with several miRNAs, including miR-140-5p, miR-181a-5p, miR-125a-5p, and miR-21-5p [156]. Together, these observations provide a plausible biological context for the association between *MEG3* variation and COPD-related phenotypes.

Certain investigated variants showed associations with lung function parameters without showing significant associations with COPD status. The associations of *MALAT1* and *SIRT3* with lung function parameters may reflect their roles in inflammation, endothelial function, mitochondrial metabolism, and oxidative stress responses [157].

The results of the polygenic score analysis suggest that genetic information is more informative when considered together with established clinical risk factors than when used alone. Both unweighted and weighted polygenic scores showed moderate discrimination when analysed separately, but substantially higher discrimination when combined with age, sex and smoking exposure. These findings are consistent with the multifactorial nature of COPD, in which genetic susceptibility acts together with cumulative environmental exposure and ageing-related biological changes. Previous studies of polygenic risk scores in COPD also support the contribution of inherited susceptibility to disease risk, lung function impairment and heterogeneity of clinical presentation. COPD polygenic risk scores derived from GWAS summary statistics for lung function traits, specifically FEV1 and FEV1/FVC from UK Biobank and SpiroMeta, have been associated not only with moderate-to-severe COPD, but also with CT-defined emphysema and airway phenotypes, indicating that genetic liability captures structural as well as spirometric components of the disease [47]. Subsequent work showed that COPD PRS was associated with earlier age at diagnosis and improved prediction of COPD before 50 years of age when incorporated into models including early-life risk factors [158]. Genome-wide COPD PRS obtained using the UK Biobank data was also shown to interact with smoking exposure, with individuals at high genetic risk being more susceptible to COPD in the presence of smoking, consistent with a gene–environment contribution to disease development [159]. More recently, addition of a COPD PRS based on UK Biobank and SpiroMeta data to the modified Lung Function Questionnaire score improved the identification of undiagnosed COPD [160]. A weighted polygenic score based on 82 signals previously identified in a COPD GWAS [161] was associated with lower lung function from childhood onwards, suggesting that COPD susceptibility may partly reflect genetically influenced lung growth as well as accelerated decline in adulthood [162]. Together, these studies are consistent with our findings, in which a weighted pathway-based polygenic score derived from COPD-associated variants improved discrimination when combined with age, sex, and smoking exposure. Although genetic information alone is unlikely to substantially reduce COPD mortality in the near future, the integration of pathway-based genetic risk scores with established clinical risk factors may facilitate earlier identification of susceptible individuals and improve risk stratification. Such approaches could support targeted preventive interventions, personalised monitoring of high-risk populations, and a better understanding of biological heterogeneity in COPD. Furthermore, the pathways identified in this study provide a framework for future functional and translational research aimed at developing novel biomarkers and therapeutic targets.

### Study Strengths and Limitations

The main strength of this study is the combined analysis of COPD risk, lung function traits, and polygenic scores in a relatively large, ethnically homogeneous study group of 747 patients with COPD and 703 controls. The use of an ethnically homogeneous study group reduced the likelihood of population stratification, while the inclusion of variants in oxidative stress, antioxidant defence, PI3K/AKT, FOXO, sirtuin, mitochondrial, and lncRNA-related pathways allowed the analysis to address mechanisms that are central to the study hypothesis. The assessment of both disease status and quantitative lung function traits made it possible to distinguish variants associated with COPD susceptibility from those potentially related to functional impairment.

The limitations of the study include the use of selected SNVs in predefined genes, which limits interpretation of the results to the examined pathways. The study sample consisted of individuals of the same ethnicity originating from the same region, which improves internal comparability but also necessitates replication in other populations. Independent validation is also needed to confirm the predictive performance of the polygenic scores. Finally, the biological effects of the associated variants require further clarification in functional experiments using relevant lung and immune-cell models.

## 5. Conclusions

The present study provides evidence that genetic variants in oxidative stress, antioxidant defence, PI3K/AKT/mTOR signalling, FOXO transcriptional regulation, mitochondrial function, and lncRNA-mediated regulatory pathways are associated with COPD susceptibility and lung function traits in the Tatar population. The combined effect of 11 COPD-associated variants, summarized as a polygenic score, was significantly associated with disease risk and improved predictive performance when integrated with age, sex, and smoking exposure. These results support the concept of COPD as a genetically influenced, ageing-related disease driven by interactions between environmental exposures, oxidative stress, cellular senescence, and impaired regulatory responses. Further replication and functional studies are needed to validate these findings and clarify their potential utility for risk prediction, biomarker development, and personalized prevention strategies.

## Figures and Tables

**Table 1:** Clinical and demographic characteristics of the COPD and control groups.

Parameters	COPD (N = 747)	Control (N = 703)	P
Age (Mean ± SD)	64.05 ± 11.16	62.77 ± 10.41	0.4310
Male (*n*, %)	669 (89.56)	614 (87.34)	0.1860
Female (*n*, %)	78 (10.44)	89 (12.66)	
BMI (Mean ± SD)	25.91 ± 5.54	26.36 ± 3.86	0.1921
Smoking status			
Non-smokers (*n*, %)	115 (15.37)	155 (22.05)	0.0025
Current and former smokers (*n*, %)	629 (84.63)	548 (77.95)	
Pack-years in smokers (Median, IQR)	40.00 (30.00–50.00)	15.00 (10.00–23.00)	0.0001
Lung function parameters			
FEV_1_/FVC (%) (Median, IQR)	59.00 (47.00–72.00)	90.08 (79.00–130.7)	0.0001
VC (%)	57.00	83.32	0.0001
(Median, IQR)	(43.00–72.10)	(62.67–92.42)	
FEV_1_ (%)	38.00	126.01	0.0001
(Median, IQR)	(28.1–54.06)	(94.75–163.80)	
FVC	56.01	125.00	0.0001
(Median, IQR)	(42.00–72.00)	(109.00–151.00)	
GOLD grades (n, %)			
GOLD I FEV_1_ ≥ 80%	0	–	–
GOLD II 50% ≤ FEV_1_< 80%	244 (32.67)		
GOLD III 30% ≤ FEV_1_< 50%	288 (38.55)		
GOLD IV FEV_1_ < 30%	214 (28.78)		
Results of the modified Medical Research Council (mMRC) dyspnoea scale questionnaire, (n, %)			
Grade 1	83 (11.11)	–	–
Grade 2	166 (22.22)		
Grade 3	341 (45.65)		
Grade 4	157 (21.02)		
Results of COPD Assessment Test (CAT) questionnaire (n, %)			
Median, IQR	23.0 (17.0–28.0)	–	–
0–10 score, (*n*, %)	58 (7.76)		
11–20 score, (*n*, %)	226 (30.25)		
21–30 score, (*n*, %)	342 (45.78)		
30–40 score, (*n*, %)	121 (16.21)		

Legend: BMI—body mass index; FEV1—forced expiratory volume in 1 s; VC—vital capacity; FVC—forced vital capacity; pack-years—number of cigarettes per day × number of years smoked/20; GOLD grades—severity of airflow obstruction in COPD based on post-bronchodilator FEV1 (https://goldcopd.org); Mean ± SD—Mean ± Standard Deviation; Median (IQR)—median and interquartile range (25th–75th percentile).

**Table 2:** Statistically significant associations between the analysed polymorphisms and chronic obstructive pulmonary disease.

Gene	SNV	EA	MAF		P_HWE_	OR (95% CI)	P	P_FDR_
			Cases	Control				
*NFE2L2*	rs6721961	G	0.27	0.33	0.109	1.39 (1.16–1.67)	3.50 × 10^−4^	0.002
*PIK3R1*	rs10515070	T	0.38	0.44	1.000	1.29 (1.07–1.56)	0.007	0.026
*PIK3R1*	rs831125	A	0.16	0.30	0.247	2.22 (1.76–2.79)	1.23 × 10^−11^	4.05 × 10^−10^
*PIK3R1*	rs3730089	A	0.35	0.42	0.217	1.31 (1.11–1.55)	0.001	0.007
*FOXO3A*	rs2253310	C	0.18	0.10	0.319	1.85 (1.47–2.35)	2.76 × 10^−7^	3.03 × 10^−6^
*GSR*	rs1002149	C	0.21	0.25	0.351	1.25 (1.04–1.48)	0.014	0.047
*CDKN2B-AS1*	rs4977574	G	0.50	0.45	0.929	1.24 (1.04–1.48)	0.016	0.048
*PTEN*	rs701848	T	0.42	0.49	1.000	1.29 (1.07–1.55)	0.007	0.026
*MEG3*	rs7158663	G	0.48	0.34	0.137	1.72 (1.45–2.05)	1.01 × 10^−9^	1.67 × 10^−8^
*TOMM40*	rs2075650	A	0.14	0.20	1.000	1.54 (1.23–1.94)	1.69 × 10^−4^	0.001
*HMOX1*	rs2071749	G	0.41	0.50	0.093	1.5 (1.26–1.77)	3.60 × 10^−6^	2.97 × 10^−5^

Legend: SNV—single-nucleotide variant; EA—effect allele; MAF—minor allele frequency; PHWE—*p*-value for the Hardy–Weinberg equilibrium test; Rec—recessive genetic model; Dom—dominant genetic model; Add—additive genetic model; OR—odds ratio; 95% CI—95% confidence interval; P—*p*-value; PFDR—*p*-value adjusted using the Benjamini–Hochberg procedure.

**Table 3:** Statistically significant associations between the analysed polymorphisms and pulmonary function parameters in patients with chronic obstructive pulmonary disease.

Trait	Gene	SNV	EA	β ± SE (95% CI)	P	P_FDR_
VC	*SIRT3*	rs3782116	G	4.11 ± 1.38 (1.41; 6.81)	0.003	0.049
VC	*NFE2L2*	rs6721961	G	−5.51 ± 1.42 (−8.30; −2.73)	1.16 × 10^−4^	0.004
FVC	*NFE2L2*	rs6721961	G	−6.73 ± 2.03 (−10.71; −2.74)	0.001	0.025
FVC	*MEG3*	rs7158663	A	6.50 ± 2.04 (2.50; 10.49)	0.002	0.025
FVC	*PIK3R1*	rs831125	A	−7.34 ± 2.52 (−12.29; −2.39)	0.004	0.042
FEV_1_/FVC	*MALAT1*	rs619586	A	8.74 ± 2.65 (3.54; 13.94)	0.001	0.035

Legend: SNV—single-nucleotide variant; EA—effect allele; Beta—effect size; SE—standard error; 95% CI—95% confidence interval; P—*p*-value; PFDR—*p*-value adjusted using the Benjamini-Hochberg procedure; VC—vital capacity; FVC—forced vital capacity; FEV1/FVC—post-bronchodilator forced expiratory volume in 1 s/forced vital capacity ratio.

**Table 4:** Comparison of net reclassification improvement between models with added unweighted and weighted polygenic scores and the baseline.

	Reference (Age + Sex + Smoking Index							
	Unweighted PRS + Age + Sex + Smoking Index				Weighted PRS + Age + Sex + Smoking Index			
	NRI	SE	95% CI	P	NRI	SE	95% CI	P
Total	0.64	0.07	0.50–0.76	2.92 × 10^−21^	0.62	0.06	0.51–0.73	1.37 × 10^−27^
Cases	0.30	0.05	0.19–0.40	4.40 × 10^−8^	0.32	0.04	0.25–0.39	3.50 × 10^−19^
Control	0.34	0.03	0.27–0.40	3.15 × 10^−25^	0.30	0.03	0.23–0.37	7.47 × 10^−20^

Legend: PRS—polygenic risk score; NRI—net reclassification improvement; SE—standard error; 95% CI—95% confidence interval; P—*p*-value.

## Data Availability

Data are contained within the article and Supplementary Materials.

## Supplementary Materials

The following supporting information can be downloaded at: https://www.mdpi.com/article/10.3390/genes17060685/s1, Table S1: Functional and regulatory annotation of the analysed genetic variants; Table S2: Genotyping methods and assay details for the analysed variants [34,35,36,37,38]; Table S3: Power calculation results; Table S4: Associations between the analysed variants and chronic obstructive pulmonary disease; Table S5: Associations between the analysed variants and quantitative phenotypes, including pulmonary function parameters.

## Abbreviations

The following abbreviations are used in this manuscript:

AKT	protein kinase B
AP-1	activator protein 1
ARE	antioxidant response element
AUC	area under the curve
Bach1	BTB and CNC homology 1
BIM	BCL-2-interacting mediator of cell death
BMI	body mass index
CAT	COPD Assessment Test
CDKN	cyclin-dependent kinase inhibitor
CI	confidence interval
COPD	chronic obstructive pulmonary disease
CT	computed tomography
EDTA	ethylenediaminetetraacetic acid
EMT	epithelial–mesenchymal transition
eQTL	expression quantitative trait locus
FDR	false discovery rate
FEV1	forced expiratory volume in 1 s
FVC	forced vital capacity
GOLD	Global Initiative for Chronic Obstructive Lung Disease
GPX1	glutathione peroxidase 1
GSH	glutathione
GWAS	genome-wide association study
HIF	hypoxia-inducible factor
ICD-10	International Classification of Diseases, 10th Revision
IL	interleukin
KEGG	Kyoto Encyclopedia of Genes and Genomes
KEAP1	Kelch-like ECH-associated protein 1
lncRNA	long non-coding RNA
MALAT1	metastasis-associated lung adenocarcinoma transcript 1
MAP1LC3B (LC3B)	microtubule-associated proteins 1A/1B light chain 3B
mMRC	Modified Medical Research Council Dyspnoea Scale
mTOR	mechanistic target of rapamycin
NADPH	nicotinamide adenine dinucleotide phosphate
NF-κB	nuclear factor kappa B
NQO1	NAD(P)H quinone dehydrogenase 1
NRF2	nuclear factor erythroid 2-related factor 2
NRI	net reclassification improvement
OR	odds ratio
PCR-RFLP	polymerase chain reaction–restriction fragment length polymorphism
PFDR	false discovery rate-adjusted *p*-value
PI3K	phosphoinositide 3-kinase
PINK1	PTEN-induced kinase 1
PRC	polycomb repressive complex
PRS	polygenic risk score
PTEN	phosphatase and tensin homolog
ROC	receiver operating characteristic
ROS	reactive oxygen species
SASP	senescence-associated secretory phenotype
SD	standard deviation
SE	standard error
SHIP1	SH2 domain-containing inositol 5-phosphatase 1
SIRT	sirtuin
SNV	single-nucleotide variant
SOD2	superoxide dismutase 2
TGF-β	transforming growth factor beta
TFBS	transcription factor binding site
VC	vital capacity
Wnt	Wingless/Integrated signalling pathway
